# Pituitary neuroendocrine tumors with PIT1/SF1 co-expression show distinct clinicopathological and molecular features

**DOI:** 10.1007/s00401-024-02686-1

**Published:** 2024-01-16

**Authors:** Matthias Dottermusch, Alice Ryba, Franz L. Ricklefs, Jörg Flitsch, Simone Schmid, Markus Glatzel, Wolfgang Saeger, Julia E. Neumann, Ulrich Schüller

**Affiliations:** 1 Institute of Neuropathology, University Medical Center Hamburg-Eppendorf, Martinistr. 52, 20246 Hamburg, Germany; 2 Center for Molecular Neurobiology (ZMNH), University Medical Center Hamburg-Eppendorf, Hamburg, Germany; 3 Department of Neurosurgery, University Medical Center Hamburg-Eppendorf, Hamburg, Germany; 4 Department of Neuropathology, Charité-Universitätsmedizin Berlin, corporate member of Freie Universität Berlin and Humboldt-Universität zu Berlin, Berlin, Germany; 5 Institute of Pathology, University Medical Center Hamburg-Eppendorf, Hamburg, Germany; 6 Pediatric Hematology and Oncology, University Medical Center Hamburg-Eppendorf, Hamburg, Germany; 7 Children’s Cancer Research Center Hamburg, Hamburg, Germany

**Keywords:** Pituitary, PitNET, PIT1, SF1, Multilineage, Methylation

## Abstract

Pituitary neuroendocrine tumors (PitNETs) are classified according to cell lineage, which requires immunohistochemistry for adenohypophyseal hormones and the transcription factors (TFs) PIT1, SF1, and TPIT. According to the current WHO 2022 classification, PitNETs with co-expression of multiple TFs are termed “plurihormonal”. Previously, PIT1/SF1 co-expression was prevailingly reported in PitNETs, which otherwise correspond to the somatotroph lineage. However, little is known about such tumors and the WHO classification has not recognized their significance. We compiled an in-house case series of 100 tumors, previously diagnosed as somatotroph PitNETs. Following TF staining, histopathological features associated with PIT1/SF1 co-expression were assessed. Integration of in-house and publicly available sample data allowed for a meta-analysis of SF1-associated clinicopathological and molecular features across a total of 270 somatotroph PitNETs. The majority (74%, 52/70) of our densely granulated somatotroph PitNETs (DGST) unequivocally co-expressed PIT1 and SF1 (DGST-PIT1/SF1). None (0%, 0/30) of our sparsely granulated somatotroph PitNETs (SGST) stained positive for SF1 (SGST-PIT1). Among DGST, PIT1/SF1 co-expression was significantly associated with scarce FSH/LH expression and fewer fibrous bodies compared to DGST-PIT1. Integrated molecular analyses including publicly available samples confirmed that DGST-PIT1/SF1, DGST-PIT1 and SGST-PIT1 represent distinct tumor subtypes. Clinicopathological meta-analyses indicated that DGST-PIT1 respond more favorably towards treatment with somatostatin analogs compared to DGST-PIT1/SF1, while both these subtypes show an overall less aggressive clinical course than SGST-PIT1. In this study, we spotlight that DGST with co-expression of PIT1 and SF1 represent a common, yet underrecognized, distinct PitNET subtype. Our study questions the rationale of generally classifying such tumors as “plurihormonal”, and calls for a refinement of the WHO classification. We propose the term “somatogonadotroph PitNET”.

**Supplementary Information:**

The online version contains supplementary material available at 10.1007/s00401-024-02686-1.

## Introduction

Pituitary neuroendocrine tumors (PitNETs), formerly termed pituitary adenomas, are common intracranial neoplasms, which originate from adenohypophyseal cells of the anterior pituitary lobe. PitNET classification is based on cell lineage, determined by immunohistochemistry for adenohypophyseal hormones and the pituitary transcription factors (TFs) PIT1, SF1, and TPIT [2, 28]. According to the current WHO classification of 2022, immunopositivity for one of these TFs denotes the PIT1-, gonadotroph, or corticotroph lineage, respectively. PitNETs of the PIT1-lineage can further exhibit somatotroph, mammosomatotroph, lactotroph and/or thyrotroph differentiation. Some PitNETs may express multiple adenohypophyseal hormones and/or TFs, belonging to more than one cell lineage. Currently, the WHO classification subsumes all such tumors under the term “plurihormonal PitNET/adenoma” [31].

First reports on PitNETs demonstrating simultaneous expression of multiple hormones date back to over 40 years ago [9, 22]. In particular, expression of gonadotropins was recurrently described in growth hormone (GH)-secreting PitNET/adenomas causing acromegaly [9, 12, 14, 22], suggesting affiliations with both the somatotroph and gonadotroph lineage in a subset of such tumors.

Through the routine implementation of TFs in pituitary diagnostics, multilineage PitNETs regained increased attention over the last few years. Several studies have reported concurrent PIT1 and SF1 immunopositivity in unusual PitNETs, which otherwise reflected somatotroph tumors [3, 16, 24, 29]. Previous transcriptome-based studies confirmed occasional SF1 expression in somatotroph PitNETs, predominantly in the densely granulated subtype [17, 20]. Moreover, SF1 expression was recently linked to distinct transcriptomic signatures and methylation profiles among somatotroph PitNETs [11, 20]. In summary, the current literature suggests that PIT1/SF1 co-expression associates with the somatotroph lineage. This finding is, however, yet underappreciated by the WHO classification and challenges the precept of declaring any and all PitNETs with co-expression of multiple TFs as “plurihormonal”. Currently, little is known about the clinical, histopathological, and molecular features linked to PIT1/SF1 co-expression in somatotroph PitNETs and such tumors are considered rare.

In this study, we aimed to demonstrate the prevalence of PIT1/SF1 co-expression in PitNETs, which otherwise correspond to somatotroph PitNETs, as defined by the WHO 2022 classification. We further explored the clinical, histopathological, epigenomic and genomic features associated with PIT1/SF1 co-expression in somatotroph tumors in a large meta-analysis by integrating molecular in-house data and publicly available data deposits.

## Methods

### Case series and tissue assembly

The archive of the University Medical Center Hamburg-Eppendorf was searched for PitNET/adenomas diagnosed between 2017 and 2023 as either sparsely or densely granulated somatotroph PitNET/adenomas or plurihormonal PitNET/adenomas. PitNETs fulfilling the essential diagnostic criteria for sparsely or densely granulated somatotroph PitNETs, as defined by the WHO 2022 Classification of Endocrine and Neuroendocrine Tumours (5th ed.) [31] were included in this study. In detail, inclusion criteria for DGST were adopted as follows: (i) diffuse GH expression (arbitrarily required in at least 30% of tumor cells), (ii) absence of other pituitary cell differentiation was disregarded owing to the purpose of this study, (iii) perinuclear LMWCK staining (perinuclear cytoplasmic CAM5.2 immunopositivity arbitrarily required in at least 30% of tumor cells), and (iv) acidophilic cytoplasm on H&E-stained sections (evaluated as either moderate or strong cytoplasmic eosinophilia). Six PitNETs were entirely immunonegative for CAM5.2 and thus did not fulfill the third WHO criterion. They were nevertheless included in the case series, due to a lack of reasonable differential diagnoses other than DGST and with the aim to further explore these unusual tumors.

Inclusion criteria for SGST were adopted as follows: (i) variable GH expression (any immunostaining pattern was accepted), (ii) absence of other pituitary cell differentiation was disregarded owing to the purpose of this study, (iii) abundant fibrous bodies (in more than 70% of cells), and (iv) absence of acidophilic cytoplasm on H&E-stained sections (evaluated as either none or weak cytoplasmic eosinophilia).

Cases of insufficient formalin-fixed paraffin-embedded (FFPE) material quantity or quality were excluded. To avoid inclusion of mammosomatotroph, mixed GH-PRL and plurihormonal PIT1-lineage tumors, cases with marked expression of estrogen receptor, prolactin or TSH were excluded from this study. Scattered intratumoral PRL expression up to 5% was tolerated for study inclusion, owing to the high prevalence of this finding, the possibility that PRL expression may stem from intratumorally entrapped residual adenohypophyseal cells and the aim to further explore this feature.

The use of all tissue specimens for research upon anonymization was in accordance with local and national ethical standards and with the 1964 Helsinki declaration and its later amendments.

### Histology and immunohistochemistry

FFPE tissue samples were sectioned into 2 µm thick slices, according to standard laboratory protocols. Immunohistochemical stainings were performed on an automated staining machine (Ventana BenchMark TX, Roche Diagnostics, Mannheim, Germany). The following primary antibodies were used: SF1 (ab217317, Abcam, 1:250), TPIT (AMAb91409, Atlas antibodies, 1:1000), PIT1 (ab272639, Abcam, 1:1000), GH (ABIN6950857, antibodies-online, 1:100), PRL (ab11301, Abcam, 1:1000), TSH (MS1453-P, Thermo scientific, 1:10,000), alpha-subunit (bs-1912R, Bioss Antibodies, 1:1000), CAM5.2 (AB_2800363, BD Biosciences, 1:1000), FSH (M3504, Dako, 1:200), LH (M3502, Dako, 1:100), Ki67 (SP6, Cell Marque, 1:750). Detection was performed with secondary antibodies and diaminobenzidine (DAB) as a chromogen. PitNETs were considered either positive or negative for TFs. No equivocal cases were encountered. A tissue microarray of non-pituitary NETs was kindly provided by Guido Sauter (Institute of Pathology, University Medical Center Hamburg-Eppendorf, Hamburg, Germany).

### DNA methylation analysis

DNA was isolated from FFPE tissue using the ReliaPrep™ FFPE gDNA Miniprep System (Promega). Around 100–500 ng of DNA was bisulfite-converted using the EZ DNA Methylation Kit (Zymo Research). The DNA Clean & Concentrator-5 kit (Zymo Research) and the Infinium HD FFPE DNA Restore Kit (Illumina) were used to clean and restore the converted DNA. Finally, the Illumina Infinium Methylation EPIC BeadChip Kit was used to quantify the methylation status of 850,000 CpG sites on an iScan device (Illumina).

Raw methylation array data (idat files) from this study and publicly available deposits [Capper et al. (GSE109381) [6]; Neou et al. (E-MTAB-7762) [17], Kober et al. (GSE226764) [11], and Silva-Júnior et al. (GSE207937) [23]] were processed using the minfi package [1] in R [19]. Probes on sex chromosomes, probes with a detection *p* value of or above 0.01, probes with SNPs at the CpG site, and cross-reactive probes were excluded. When combining different types of arrays, probes, which were not represented in both the EPIC and the 450 k array were excluded.

Consensus cluster analyses were performed using the ConsensusClusterPlus package [32]. Uniform Manifold Approximation and Projection (UMAP) transformation and plotting were performed using the umap package [15].

Cumulative copy number profiles (CNPs) were calculated using the GenVisR package [26]. To reduce CNP noise, three consecutive segments were required to surpass the cutoffs set to − 0.35 and 0.35.

Methylation-based classification was performed using the R package of the MNP brain tumor methylation classifier v12.5 [6].

### Data integration and annotation of external samples

For evaluation of clinicopathological parameters in DGST-PIT1/SF1, DGST-PIT1, and SGST-PIT1 across studies, we compiled an extended case series consisting of our in-house samples, and publicly available data of somatotroph PitNETs derived from Capper et al. [6] (GSE109381), Neou et al. [17] (E-MTAB-7762, E-MTAB-7768), Silva-Júnior et al. [23] (GSE207937, GSE209903), Kober et al. [11] (GSE226764) and Rymuza et al. [20] (E-MTAB-11889). Extended sample data of the latter study were kindly provided by Mateusz Bujko (Dept. of Molecular and Translational Oncology, Maria Sklodowska-Curie National Research Institute of Oncology, Warsaw, Poland). For preparation of the integrated dataset, in-house samples were classified as either DGST-PIT1/SF1, DGST-PIT1, or SGST-PIT1, based on histomorphology and SF1-IHC. External samples were reclassified according to *NR5A1* (SF1) RNA expression levels and/or methylation classifier results. Normalized *NR5A1* counts above or below 160 were considered high, or low, respectively. External samples of Rymuza et al. lacked both DNA methylation and RNA expression data and were reclassified according to the previously described qPCR-based transcriptomic subgroups [20]. To maintain reclassification accuracy, samples of Rymuza et al. were excluded if histomorphology was not in line with the qPCR-based transcriptomic subgroup.

Due to the purpose of this study, all external sample with a classifier match or histomorphology other than somatotroph PitNET (e.g., mixed GH/PRL) were excluded. Furthermore, samples demonstrating an inconclusive mismatch between SF1 status and classifier result, as well as insufficient data for accurate reclassification were excluded. The final extended case series comprised a total of 270 tumors (99 in-house, 38 cases from Capper et al., 43 cases from Kober et al., 23 cases from Neou et al., 59 cases from Rymuza et al., 8 cases from Silva-Júnior et al.).

### Statistical analyses

Statistical analyses of clinicopathological parameters were performed using the stats R package (v4.1.3) [19]. Continuous scale data were analyzed using the student’s *t* test. Ranked/ordinal data were analyzed using the Wilcoxon rank sum test. Binary categorical data were analyzed using the chi-square test. For detection of significant recurrent chromosomal alterations, the Genomic Identification of Significant Targets in Cancer (GISTIC) procedure [4] was applied using the CNVRanger R package [25]. Population ranges were computed based on reciprocal overlap between genomic regions with a cutoff of 0.51. In order to increase specificity for significant recurrent chromosomal alterations, at least 5 Mbp of genomic regions were required to show *p* values < 0.05 for each chromosome.

## Results

We searched the archive of the University Medical Center Hamburg-Eppendorf for PitNETs, which fulfilled the essential diagnostic criteria for densely (DGST) or sparsely granulated somatotroph PitNET (SGST) as defined by the WHO 2022. Owing to the purpose of this study, hormone, and TF expression outside of the PIT1-lineage were overlooked as exclusion criteria.

### PIT1/SF1 co-expression is highly prevalent in DGST

**Fig. 1:**
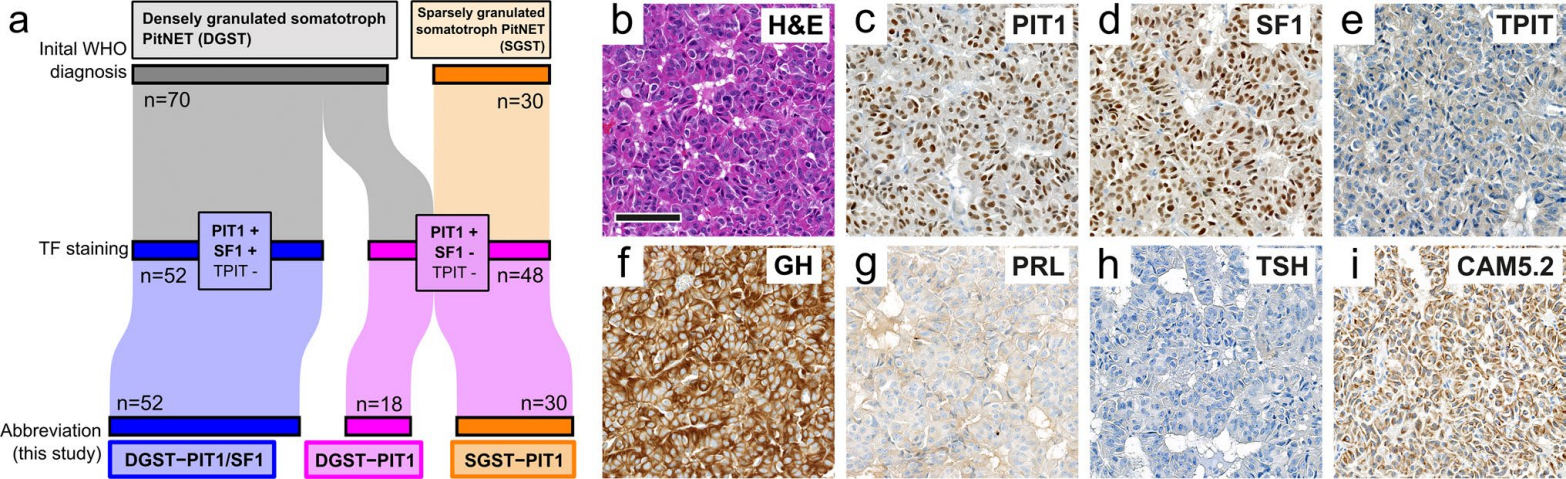
Densely granulated somatotroph PitNETs frequently co-express PIT1 and SF1. **a** Sankey diagram demonstrates the occurrence of PIT1/SF1 co-expression among in-house samples of densely and sparsely granulated somatotroph PitNETs (DGST and SGST, respectively). PIT1/SF1 co-expression was restricted to most DGST (subsequently termed DGST-PIT1/SF1). The remaining tumors exclusively expressed PIT1 (subsequently termed DGST-PIT1 or SGST-PIT1). **b-**i Microscopic images of the histomorphology and immunostaining profile of a representative DGST-PIT1/SF1. This tumor showed strongly eosinophilic cytoplasm (B) and co-expression of PIT1 (**c**) and SF1 (**d**). No staining for TPIT (**e**) was detected. Growth hormone (GH) was strongly expressed (**f**), while prolactin (PRL, **g**) and thyroid-stimulating hormone (TSH, **h**) were immunonegative. CAM5.2 predominantly stained the perinuclear cytoplasm (**i**). Scale bar is 100 µm in **b**-i

A total of 100 PitNETs were assembled (Supplementary Table 1). The series comprised 70 DGST (64 of which had been previously diagnosed as DGST, 6 of which had also been termed “plurihormonal” due to detection of FSH, LH and/or SF1 expression during diagnostic workup) and 30 SGST (all previously diagnosed as SGST). The 100 PitNETs were stained for PIT1, SF1, and TPIT. The vast majority of DGSTs displayed strong and unequivocal co-expression of PIT1 and SF1 (52/70 (74.3%), Fig. 1a–i, subsequently referred to as DGST-PIT1/SF1). The remaining DGSTs demonstrated nuclear staining for PIT1 and were clearly void of SF1 expression (18/70 (25.7%), subsequently referred to as DGST-PIT1, Fig. 1a). All 30 SGST (subsequently referred to as SGST-PIT1) showed strong nuclear staining for PIT1 and were clearly immunonegative for SF1 (Fig. 1a). None of the 100 PitNETs (i.e., DGST-PIT1/SF1, DGST-PIT1 and SGST-PIT1) expressed TPIT. Sensitivity and specificity of our TF antibodies was validated by appropriate and expected staining results of further non-PIT1 lineage PitNETs (*n* = 60; Supplementary Fig. 1a–h) [2] and non-pituitary NETs (*n* = 151; Supplementary Fig. 1i) [30].

### Histopathological feature comparison

**Fig. 2:**
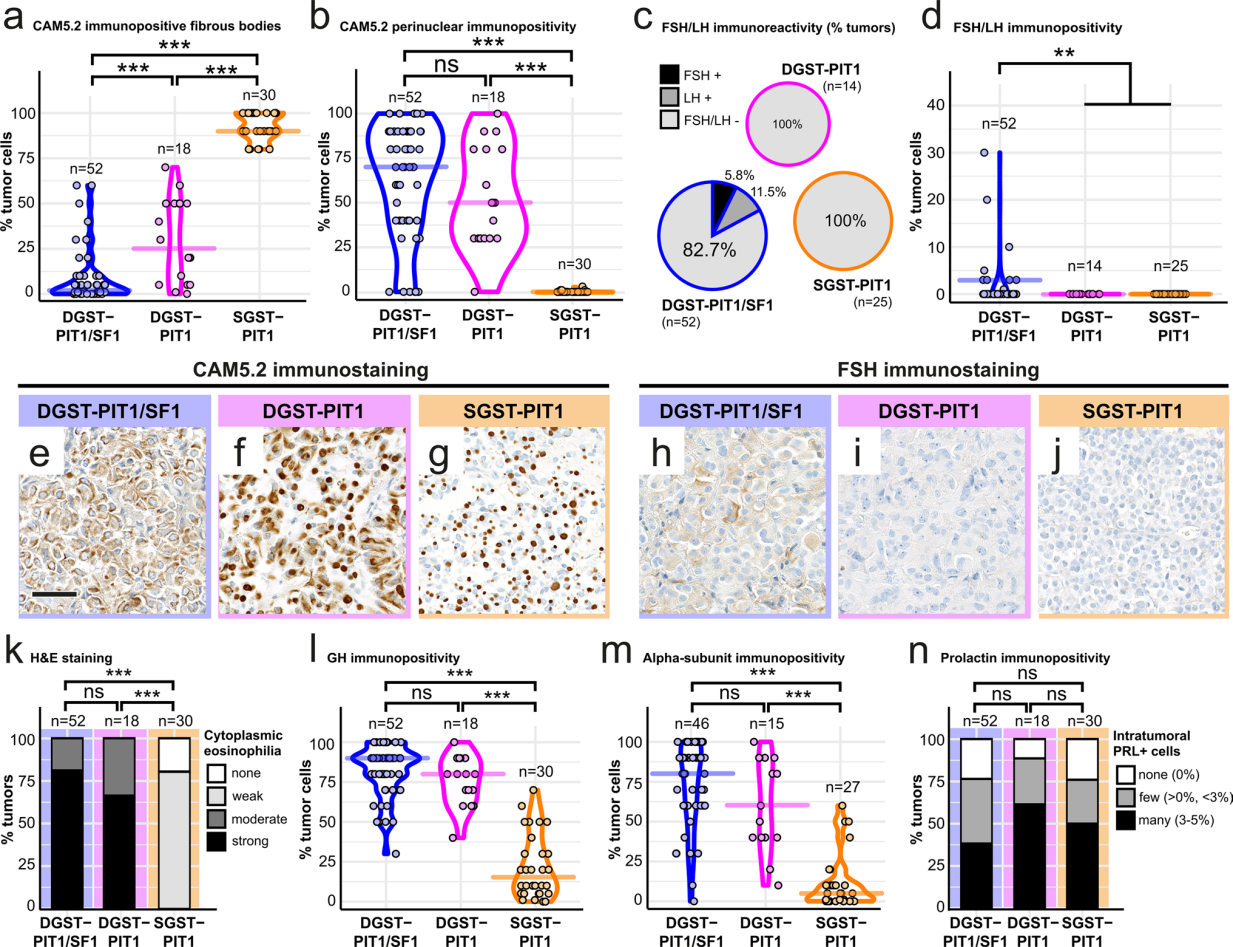
Comparison of histopathological features in DGST-PIT1/SF1, DGST-PIT1 and SGST-PIT1. **a**, **b** CAM5.2 immunostaining demonstrated that DGST-PIT1/SF1 harbor significantly less fibrous bodies (median: 3%) compared to DGST-PIT1 (median: 25%, *p* < 0.001) and SGST-PIT1 (median: 90%, *p* < 0.001) (**a**). Perinuclear cytoplasmic CAM5.2 immunoreactivity was similar in both DGST-PIT1/SF1 (median: 70%) and DGST-PIT1 (median: 50%) and significantly increased compared to SGST-PIT1 (median: 0%, *p* < 0.001) (**b**). Wilcoxon rank sum test. **c**, **d** Nine of 52 (17.3%) DGST-PIT1/SF1 demonstrated expression of FSH [3/52 (5.8%)] or LH [6 /52 (11.5%)]. Median of FSH/LH immunopositive tumor cells among FSH/LH-expressing DGST-PIT1/SF1 was 3% (range: 1–30%). Wilcoxon rank sum test. **e**–**g** CAM5.2 immunoreactivity is demonstrated in a representative DGST-PIT1/SF1 (**e**), DGST-PIT1 (**f**) and SGST-PIT1 (**g**). **h**–**j** FSH immunoreactivity is demonstrated in a representative DGST-PIT1/SF1 (**h**), DGST-PIT1 (**i**) and SGST-PIT1 (**j**). Scale bar is 50 µm in (**e**–**j**). **k** Cytoplasmic eosinophilia was strong or moderate in DGST-PIT1/SF1 (42 (81%) and 10 (19%) of 52, respectively) and DGST-PIT1 [12 (67%) and 6 (33%) of 18, respectively] with no obvious differences between the two groups (*p* = 0.22). SGST-PIT1 showed either weak or no eosinophilia (24 (80%) and 6 (20%) of 30, respectively). Wilcoxon rank sum test. l) GH expression was highly prevalent in DGST-PIT1/SF1 (median: 90%) and DGST-PIT1 (median: 80%) with no significant differences between the two (*p* = 0.09). SGST-PIT1 showed rather low GH expression (median: 15%, *p* < 0.001 compared to both DGST-PIT1/SF1 and DGST-PIT1). Wilcoxon rank sum test. m) Alpha-subunit expression was high in DGST-PIT1/SF1 (median: 80%) and DGST-PIT1 (median: 60%) with no significant differences between the two (*p* = 0.11). SGST-PIT1 showed rather low alpha-subunit expression (median: 5%, *p* < 0.001 compared to both DGST-PIT1/SF1 and DGST-PIT1). Wilcoxon rank sum test. n) Intratumoral prolactin expression was found in DGST-PIT1/SF1 (40/52, 77%), DGST-PIT1 (16/18, 89%) as well as SGST-PIT1 (23/30, 77%). There were no statistically significant differences between the groups (*p* = 0.09 DGST-PIT1/SF1 vs. DGST-PIT1; *p* = 0.48 DGST-PIT1/SF1 vs. SGST-PIT1; *p* = 0.35 DGST-PIT1 vs. SGST-PIT1). Wilcoxon rank sum test. ***p < 0.001, **p < 0.01, *p ≤ 0.05, *p* > 0.05 ns

We continued to compare histopathological features of DGST-PIT1/SF1, DGST-PIT1, and SGST-PIT. As expected, CAM5.2 immunopositive fibrous bodies were highly abundant in SGST-PIT1 (median 90% of tumor cells, compared to 3% in DGST-PIT1/SF1 and 25% in DGST-PIT1, *p* < 0.001; Fig. 2a, e–g), whereas DGST-PIT1/SF1 and DGST-PIT1 displayed more prominent cytoplasmic CAM5.2 immunoreactivity (median 70% and 50%, respectively, compared to 0% in SGST-PIT1; *p* < 0.001 ***; Fig. 2b, e–g). Comparisons within the group of DGST revealed that fibrous bodies were significantly less prevalent in DGST-PIT1/SF1 compared to DGST-PIT1 (*p* < 0.001), whereas the extend of cytoplasmic CAM5.2 staining was similar in both groups (*p* = 0.10 ns).

Since SF1 is a marker of the gonadotroph lineage, we assessed expression of the gonadotropins FSH and LH. Only 9 of 52 (17.3%) DGST-PIT/SF1 expressed FSH or LH, whereas immunopositivity for these hormones was not found in DGST-PIT1 (0/14) or SGST-PIT1 (0/25) (Fig. 2c, h–j). Overall expression levels were rather low among FSH/LH-positive DGST-PIT1/SF1 with a median of 3% (range: 1–30%) immunopositive tumor cells (Fig. 2d, h–j). Based on the WHO 2022 diagnostic criteria for somatotroph tumors, we moreover assessed cytoplasmic eosinophilia, growth hormone (GH) expression, and alpha-subunit expression. As expected, both DGST-PIT1/SF1 and DGST-PIT1 showed strong eosinophilia (Fig. 2k), GH expression (Fig. 2l) and alpha-subunit expression (Fig. 2m) compared to SGST-PIT1 (*p* < 0.001 in all three analyses). We discovered no significant differences between DGST-PIT1/SF1 and DGST-PIT1 in these three analyses (*p* = 0.22, *p* = 0.09, *p* = 0.11, respectively). Of note, intratumoral prolactin (PRL) expression was found in the majority of PitNETs (40/52 (77%) in DGST-PIT1/SF1, 16/18 (89%) in DGST-PIT1, 23/30 (77%) in SGST-PIT1) with insignificant differences between the three groups (Fig. 2n). No tumors stained positive for TSH (0/52 DGST-PIT1/SF1, 0/18 DGST-PIT1, 0/30 SGST-PIT1). In summary, histopathological investigations comparing DGST-PIT1/SF1 and DGST-PIT1 revealed that DGST-PIT1/SF1 show infrequent and scarce expression of FSH or LH and harbor less fibrous bodies than DGST-PIT1.

### Molecular feature comparison

We continued to investigate global DNA methylation profiles in a subset of our series (31/100 cases, including 14 DGST-PIT1/SF1, 12 DGST-PIT1, and 5 SGST-PIT1). Samples were selected based on tissue availability and histopathological features of interest. In detail, this subset included 24 PitNETs with intratumoral prolactin expression, 4 DGST lacking CAM5.2 immunoreactivity, and 3 DGST-PIT1/SF1 with FSH/LH expression.

**Fig. 3:**
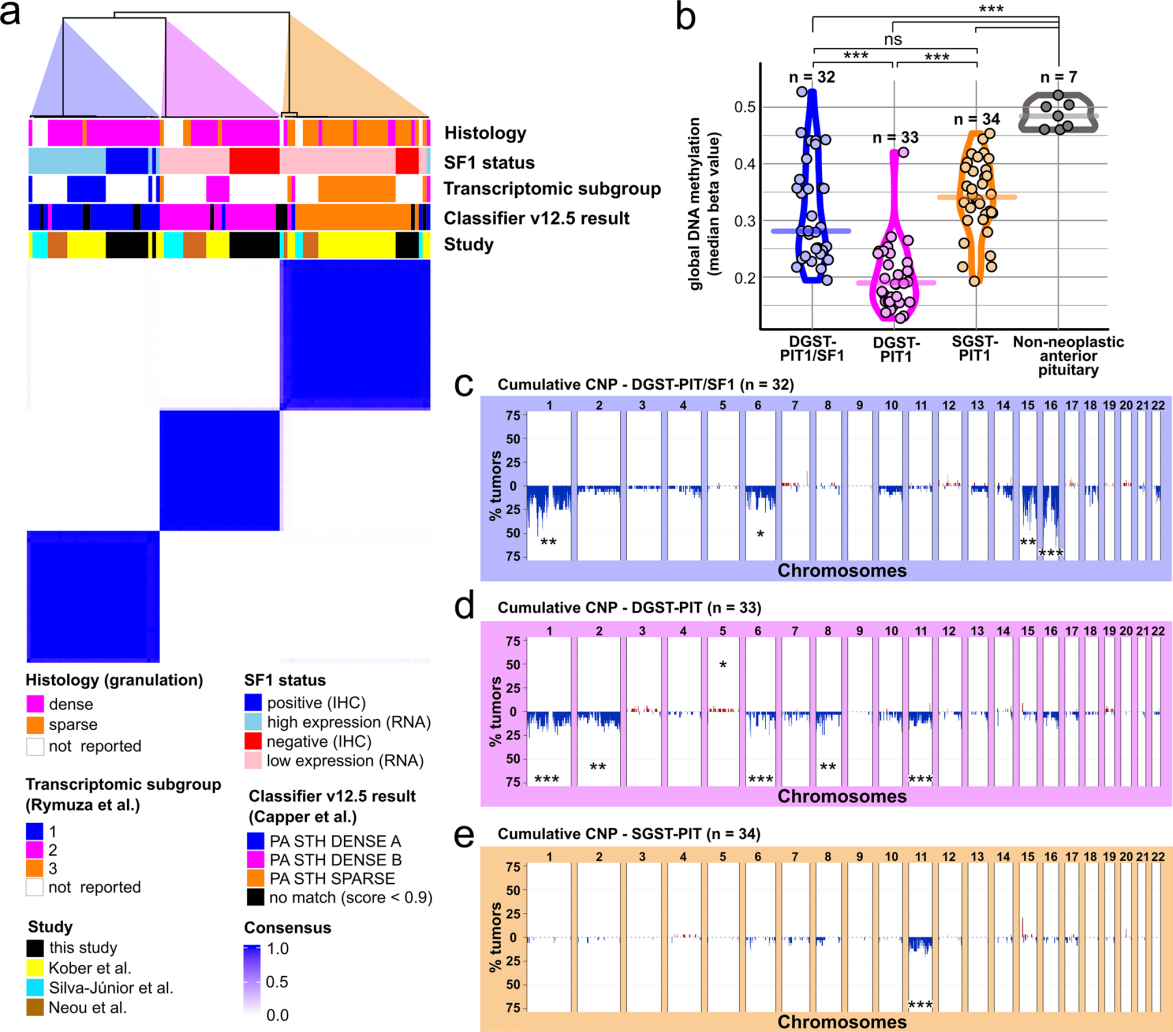
Comparison of molecular features in DGST-PIT1/SF1, DGST-PIT1 and SGST-PIT1. **a** Consensus clustering of global DNA methylation data demonstrated three epigenetically distinct subgroups of somatotroph tumors. The first subtype (left branch) mostly harbored tumors with densely granulated histology and evident SF1-expression via RNA or immunostaining, showed an affiliation with the transcriptomic subgroup 1 (defined by Rymuza et al. [20]), and matched with the methylation class (mc) “Pituitary adenoma, subtype STH-producing, subclass densely granulated A” (PA STH DENSE A) (Brain classifier version 12.5). The second subtype (middle branch) mostly harbored tumors with densely granulated histology and insignificant SF1-expression via RNA or immunostaining, showed an affiliation with the transcriptomic subgroup 2, and matched with the mc PA STH DENSE B. The third subtype (right branch) mostly harbored tumors with sparsely granulated histology and insignificant SF1-expression via RNA or immunostaining, showed an affiliation with the transcriptomic subgroup 3, and matched with the mc PA STH SPARSE. Taken together, the three epigenetic subgroups correspond to DGST-PIT1/SF1 (left), DGST-PIT1 (middle), and SGST-PIT1 (right). Consensus clustering was based on the beta values of the top 1‰ variant CpG sites, *k* = 3 is shown. **b** Global DNA methylation levels were low in somatotroph PitNETs compared to anterior pituitary control tissue. DNA hypomethylation was particularly prominent in DGST-PIT1 with a median beta value of 0.19 in DGST-PIT1 compared to 0.28 in DGST-PIT1/SF1 (*p* < 0.001), 0.34 in SGST-PIT (*p* < 0.001), and 0.48 in adenohypophyseal control tissue (*p* < 0.001). ***p < 0.001, *p* > 0.05 ns, Student’s *t* test. **c**–**e** Cumulative copy number profiles (CNPs) revealed recurrent genomic aberrations in DGST-PIT1/SF1 involving losses of chromosomes 1, 6, 15 and 16 (**c**). In comparison, DGST-PIT1 exhibited occasional gains in chromosome 5 and occasional losses of chromosomes 1, 2, 6, 8, and 11 (**d**). SGST-PIT1 CNPs were rather quiet, with occasional losses of chromosome 11 (**e**). Asterisks indicate significant recurrent chromosomal alterations determined by the GISTIC procedure. *p* values of the most significantly altered genomic region are demonstrated for each chromosome: ***p < 0.001, **p < 0.01, *p ≤ 0.05. Unmarked chromosomes did not exhibit recurrent alterations designated as significant

The methylation data of our 31 in-house samples were integrated into a large reference series of 77 somatotroph PitNETs compiled from three independent publicly available datasets [11, 17, 23]. Integrated methylation data consensus cluster analysis confirmed that somatotroph tumors separate into three distinct epigenomic subclusters, as recently described (Fig. 3a) [11]. SF1 expression depicted by immunoreactivity (this study) and RNA expression levels (external studies) demonstrated that the three epigenomic subclusters relate to the tumors denoted as DGST-PIT1/SF1, DGST-PIT1 and SGST-PIT1 in this study. The three previously described transcriptome-based molecular somatotroph subgroups of Rymuza et al. [20] adequately affiliated with these three clusters (subgroups 1, 2 and 3, conformed to DGST-PIT1/SF1, DGST-PIT1 and SGST-PIT1, respectively). Using the DKFZ brain tumor methylation classifier (www.molecularneuropathology.org; v12.5) [6], DGST-PIT1/SF1 mainly matched with the methylation class (mc) “pituitary adenoma, subtype STH-producing, subclass densely granulated A (novel)” (“DNS-A”), while DGST-PIT matched with the mc “pituitary adenoma, subtype STH-producing, subclass densely granulated B (novel)” (“DNS-B”). SGST-PIT1 matched with the mc “pituitary adenoma, subtype STH-producing, subclass sparsely granulated (novel)” (“SPAR”).

In addition, we investigated how epigenomic profiles of somatotroph PitNETs associate with intratumoral prolactin expression, CAM5.2 immunonegativity and FSH/LH-expression (Supplementary Fig. 2a–f). UMAP analyses indicated that intratumoral PRL expression was not linked to epigenomic distinctness among any subtypes of somatotroph PitNETs (Supplementary Fig. 2d). The extent of cytoplasmic CAM5.2 immunoreactivity also had no impact on the clustering of DGST (Supplementary Fig. 2e). Moreover, FSH/LH expression was not associated with epigenomic distinctness among DGST-PIT1/SF1 (Supplementary Fig. 2f).

We continued to investigate global DNA methylation levels of PitNETs, reclassified as either DGST-PIT1/SF1, DGST-PIT1 or SGST-PIT1. The three subtypes demonstrated global DNA hypomethylation compared to anterior pituitary control tissue, while this finding was particularly pronounced in DGST-PIT1 (Fig. 3b).

Next, cumulative copy number profiles (CNPs) were calculated for the three somatotroph PitNET subtypes and investigated for significant recurrent chromosomal alterations (Fig. 3c–e). We found that DGST-PIT1/SF1 frequently harbored recurrent chromosomal alterations including losses in chromosomes 1, 6, 15, and 16 (Fig. 3c). DGST-PIT1 exhibited occasional gains in chromosome 5 and losses in chromosomes 1, 2, 6, 8, and 11 (Fig. 3d). SGST-PIT1 harbored recurrent losses in chromosome 11 (Fig. 3e).

In summary, DGST-PIT1/SF1, DGST-PIT1 and SGST-PIT1 were clearly molecularly distinct in terms of epigenomic signatures and CNPs.

### Clinicopathological meta-analysis

**Fig. 4:**
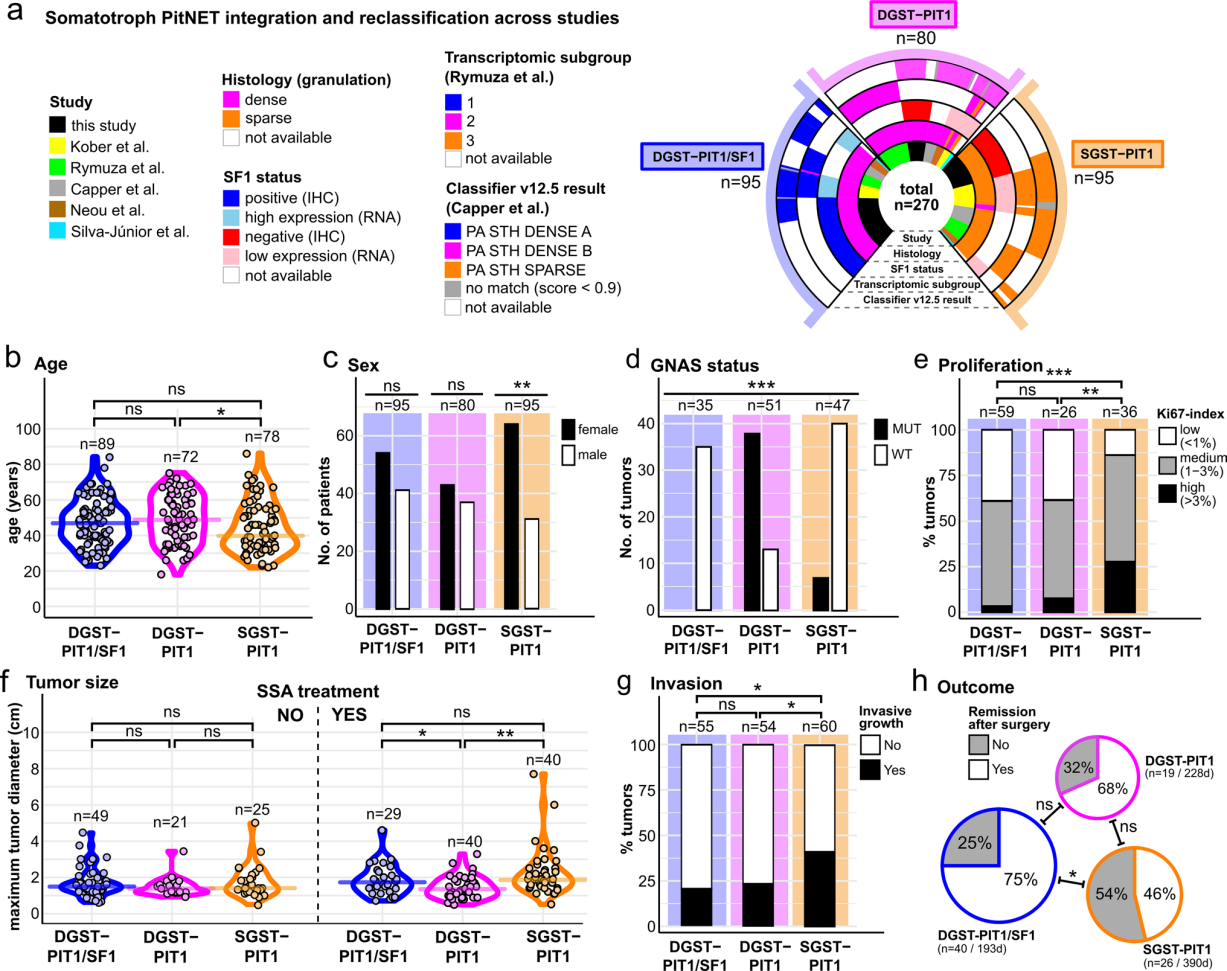
Meta-analyses of clinicopathological features in DGST-PIT1/SF1, DGST-PIT1 and SGST-PIT1. **a** A total of 270 somatotroph PitNETs, derived from multiple studies were reclassified as DGST-PIT1/SF1, DGST-PIT1 or SGST-PIT1 based on available data on histology, SF1 status, transcriptomic subgroup, and/or brain tumor classifier result. **b** Median age of patients with DGST-PIT1/SF1, DGST-PIT1 and SGST-PIT1 at time point of primary tumor resection were 47 (range: 23–84), 49 (range: 18–75), and 40 (range: 22–86), respectively. We found that patients with DGST-PIT1 were significantly older than patients with SGST-PIT1 (*p* = 0.045). Student’s *t* test. **c** Both sexes were similarly prevalent among DGST-PIT1/SF1 (F/M: 1.32, *p* = 0.24) and DGST-PIT1 (F/M: 1.16, *p* = 0.6), while SGST-PIT1 demonstrated a highly significant female predominance (F/M ratio: 2.06, *p* = 0.001). Chi-square test (expected ratio 1.0). **d** DGST-PIT1/SF1 were exclusively GNAS wild type (35/35, 100%). DGST-PIT1 harbored GNAS mutations in 38/59 (64.4%) of cases. SGST-PIT1 harbored GNAS mutations in 7/47 (15%) of cases. Chi-square test. **e** Proliferative rate determined via Ki67 immunostaining in DGST-PIT1/SF1 and DGST-PIT1 was mainly low to medium with insignificant differences between the two groups (*p* = 0.8). In comparison, proliferation in SGST-PIT1 was markedly increased (*p* < 0.001, *p* = 0.009, respectively). Wilcoxon rank sum test. f) Maximum tumor diameter medians for DGST-PIT1/SF1, DGST-PIT1 and SGST-PIT1 in patients without prior SSA treatment were 1.5 (range: 0.6–4.46), 1.4 (range: 0.92–3.43), and 1.4 (range: 0.47–5) cm, respectively. Differences between the groups were statistically insignificant (DGST-PIT1/SF1 vs. DGST-PIT1 *p* = 0.25; DGST-PIT1/SF1 vs. SGST-PIT1 *p* = 0.66¸ DGST-PIT1 vs. SGST-PIT1 *p* = 0.21). Maximum tumor diameter medians for DGST-PIT1/SF1, DGST-PIT1 and SGST-PIT1 in patients with prior SSA treatment were 1.8 (range: 0.7–4.6), 1.4 (range: 0.5–3.3), and 1.9 (range: 0.51–7.7) cm, respectively. DGST-PIT1 were significantly smaller than DGST-PIT1/SF1 (*p* = 0.039), and SGST-PIT1 (*p* = 0.001). No statistically significant difference comparing DGST-PIT1/SF1 and SGST-PIT1 (*p* = 0.13). Student’s *t* test. **g** Tumor invasiveness (defined as Knosp grade 3–4) was found in 20% of DGST-PIT1/SF1 (11/55) and 24% of DGST-PIT1 (13/54) with insignificant differences between the two groups (*p* = 0.61). In comparison, SGST-PIT1 were more frequently invasive (25/60 (42%), *p* = 0.018 and *p* = 0.048 compared to DGST-PIT1/SF1 and DGST-PIT1, respectively). Wilcoxon rank sum test. **h** Remission rate after surgery was similar in DGST-PIT1/SF1 (75%) compared to DGST-PIT1 (68%) (*p* = 0.76). Remission rate among SGST-PIT1 was 46% (*p* = 0.02 compared to DGST-PIT1/SF1 and *p* = 0.2 compared to DGST-PIT1). Median follow-up times of patients with primary tumors was 193, 228 and 390 days for DGST-PIT1/SF1, DGST-PIT1 and SGST-PIT1, respectively. Chi-square test

**Fig. 5:**
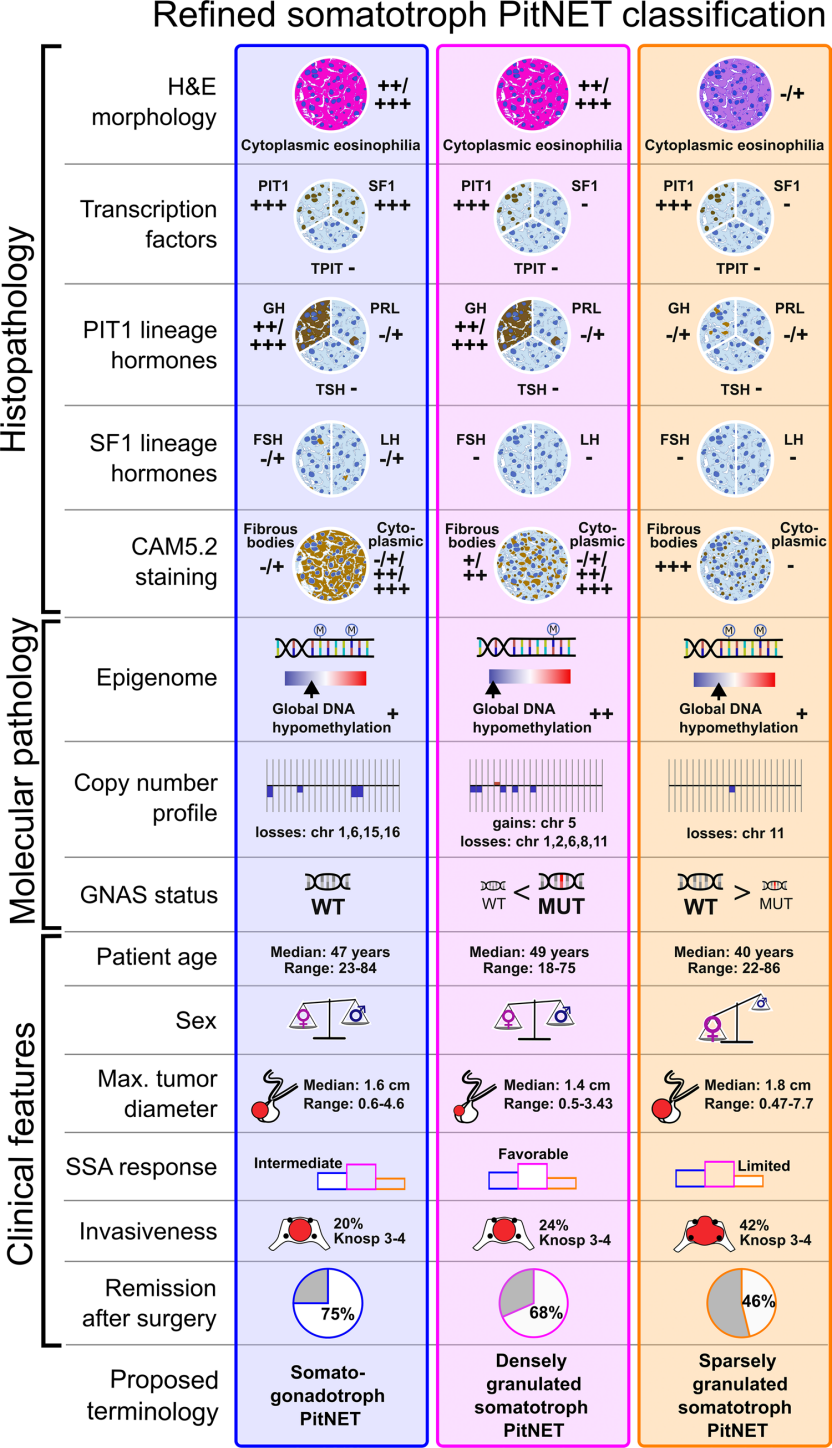
Refined somatotroph PitNET classification proposal. Overview of the histopathological, molecular, and clinical features associated with the three emerging subtypes of somatotroph PitNETs. This figure summarizes insights obtained from integrated data of this study and previous publications

In order to further explore patient and tumor characteristics of DGST-PIT1/SF1, DGST-PIT1, and SGST-PIT1 across studies in a large meta-analysis, we integrated clinicopathological data of our in-house series and public deposits of five previously published studies [6, 11, 17, 20, 23]. All samples were reclassified as either DGST-PIT1/SF1, DGST-PIT1, or SGST-PIT1 based on available histopathological, transcriptome and/or methylome data, resulting in an extended case series of 270 somatotroph tumors (95 DGST-PIT1/SF1, 80 DGST-PIT1, and 95 SGST-PIT1) (Fig. 4a). Median age of patients at primary surgery was 47 (range: 23–84), 49 (range: 18–75), and 40 (range: 22–86) years, respectively (Fig. 4b). SGST-PIT1 showed a significant female predominance (F/M ratio: 2.06, *p* = 0.001), in contrast to DGST-PIT1/SF1 (F/M: 1.32, *p* = 0.24) and DGST-PIT1 (F/M: 1.16, *p* = 0.6) (Fig. 4c). GNAS mutations are recurrent in somatotroph tumors [33], and GNAS statuses have been previously linked to the molecular somatotroph subgroups described by Rymuza et al. [20]. In the integrated data set, DGST-PIT1/SF1 were exclusively GNAS wild type (100%, 35/35), the majority of DGST-PIT1 harbored GNAS mutations (64.4%, 38/59) and most SGST-PIT1 were GNAS wild type (85%, 40/47). (Fig. 4d). SGST-PIT1 showed an increased proliferative rate compared to both DGST-PIT1/SF1 (*p* < 0.001) and DGST-PIT1 (*p* = 0.009), whereas differences in proliferation between DGST-PIT1/SF1 and DGST-PIT1 were insignificant (*p* = 0.8) (Fig. 4e). Tumor sizes were compared between the three subtypes in patients stratified by preoperative therapy with somatostatin analogs (SSA). Median maximum tumor diameters of DGST-PIT1/SF1, DGST-PIT1 and SGST-PIT1 were 1.5 (range: 0.6–4.46), 1.4 (range: 0.92–3.43), and 1.4 (range: 0.47–5) cm in patients, which had not been treated with SSA prior to surgery. No statistically significant differences were seen between the tumor groups (*p* > 0.2 in all three comparisons, Fig. 4f). Median maximum tumor diameters of DGST-PIT1/SF1, DGST-PIT1 and SGST-PIT1 were 1.8 (range: 0.7–4.6), 1.4 (range: 0.5–3.3), and 1.9 (range: 0.51–7.7) cm in patients, which had been treated with SSA prior to surgery. DGST-PIT1 were significantly smaller than DGST-PIT1/SF1 (*p* = 0.039), and SGST-PIT1 (*p* = 0.001). No statistically significant difference was seen between DGST-PIT1/SF1 and SGST-PIT1 (*p* = 0.13, Fig. 4f). Invasive growth (defined as Knosp grade 3–4) was found in 11 of 55 (20%) DGST-PIT1/SF1 and 13 of 54 (24%) DGST-PIT1 with insignificant differences between the two groups (*p* = 0.61). In comparison, invasive growth was found in 25 of 60 (42%) SGST-PIT1, which was significantly more than in DGST-PIT1/SF1 (*p* = 0.018) and DGST-PIT1 (*p* = 0.048) (Fig. 4g). In terms of clinical outcomes, patients with SGST-PIT1 showed lower rates of remission following surgical interventions (46% remission, median follow-up: 390 days), with more frequent disease persistence or relapse, compared to the similar rates of both DGST-PIT1/SF1 (75% remission, median follow-up: 193 days) and DGST-PIT1 (68% remission, median follow-up: 228 days) (Fig. 4h). Clinicopathological and molecular features of the three somatotroph PitNET subtypes are summarized in Fig. 5.

## Discussion

In this study, we aimed to explore the histopathological, molecular, and clinical features associated with the emerging role of PIT1/SF1 co-expression in PitNETs. Our study design was centered around the current WHO classification and based on findings of previous publications, which triggered the search for PIT1/SF1 co-expression among PitNETs, which otherwise correspond to the somatotroph PIT1-lineage.

### PIT1/SF1 co-expression is highly prevalent among DGST

We were surprised to find that the vast majority (nearly 3/4) of previously diagnosed DGST co-expressed PIT1 and SF1. This unexpectedly high rate can be explained by two circumstances. Firstly, the WHO has only recently dictated necessity for staining all three TFs in routine pituitary diagnostics. Thus, data on unusual TF expression constellations are currently scarce and the prevalence of multilineage PitNETs may be much higher than generally assumed. Secondly, the strict implementation of WHO-based inclusion criteria may have inflated the prevalence of PIT1/SF1 co-expression within the case series. Thus, the frequency of PIT1/SF1 co-expression among PitNETs, which do not fit into the WHO class of somatotroph tumors has yet to be determined. This pertains to pure somatotroph tumors, which do not fulfill the essential criteria for either SGST or DGST, to somatotroph tumors exhibiting elements of mammosomatotroph, mixed somatotroph-lactotroph, or PIT1-plurihormonal differentiation and to further non-somatotroph PitNETs of the PIT1-lineage.

### DGST-PIT1/SF1 exhibit distinct molecular, histopathological, and clinical features

Global DNA methylation patterns are considered to reflect the cell of origin, making epigenomic analyses useful for classifying tumors based on their lineage [6, 7, 21]. We compared methylation profiles of DGSTs with and without PIT1/SF1 co-expression and showed that they are epigenomically distinct. This result is in line with a recent publication by Kober et al. [11] and compatible with the notion that transcription factors play an early role in pituitary lineage development. The existence of two epigenomically distinct groups of tumors among DGST has also been described before by Capper et al. [6], and was implemented into the Brain tumor methylation classifier. The two groups had been termed mc “DNS-A” and mc “DNS-B” (v11b4, v12.5, v12.8), while their significance remained unknown. In this report, we clarify that the mc “DNS-A” affiliates with PIT1/SF1 co-expression, whereas the mc “DNS-B” mainly comprises pure PIT1-lineage tumors.

Moreover, we show that histopathological features of most DGST-PIT1/SF1 reflect those of prototypical DGST. In contrast, DGST-PIT1 display fibrous bodies in significantly higher amounts than DGST-PIT1/SF1. We conclude that DGST-PIT1 predominantly pertain to the previously proposed class of “intermediate type” granulated somatotroph PitNET, which were considered within the histomorphological spectrum of DGST [18]. Although “intermediate type” granulation was not exclusively encountered among DGST-PIT1, our results show that this granulation type associates with molecular distinctness from somatotroph PitNETs with densely granulated morphology.

Tumor sizes in DGST-PIT1 were significantly smaller than in DGST-PIT1/SF1 and SGST-PIT1 after SSA treatment, indicating that tumor growth of DGST-PIT1 is more efficiently impeded by medical management compared to the other subtypes. In line with this, GNAS mutations and dense granulation patterns were previously linked to favorable SSA responses in somatotroph PitNETs [5, 8, 13, 34]. We find that both these features affiliate with DGST-PIT1.

### Somatotroph PitNETs frequently demonstrate intratumoral PRL expression and may be CAM5.2 immunonegative

Handling apparent intratumoral PRL expression in somatotroph PitNETs poses a diagnostic difficulty. Firstly, it can be challenging to histomorphologically differentiate if scarce immunosignal stems from entrapped non-neoplastic pituitary cells or scattered tumor cells. Secondly, in contrast to the WHO 2017, the WHO 2022 states that somatotroph tumors are negative for PRL, raising the question how to classify bona fide somatotroph tumors with little, yet obvious intratumoral PRL expression. In this study, intratumoral PRL expression up to 5% was tolerated for somatotroph PitNET diagnosis. We found that intratumoral PRL expression did not relate to epigenomic distinctness in somatotroph PitNETs. This result clearly showcases that detection of limited PRL expression does not justify exclusion of somatotroph PitNET diagnosis. This should be considered by the WHO classification, which currently states that absence of hormone expression other than GH is an essential diagnostic criterion for somatotroph PitNETs. To further clarify this topic, extended and comprehensive epigenomic investigations on the various histopathological subtypes of PIT1-lineage PitNETs are needed.

Moreover, we epigenomically analyzed four CAM5.2 immunonegative somatotroph PitNETs in our case series. Because CAM5.2-negativity poses a diagnostic dilemma, previous studies refrained from classifying such tumors as sparsely or densely granulated [10, 27]. The epigenomic data presented in this study suggest that lack of CAM5.2 immunoreactivity among DGST does not accompany epigenomic distinctness. In addition, the highly variant extent of cytoplasmic CAM5.2 immunoreactivity (ranging from 30 to 100% of tumor cells) in the CAM5.2-positive DGST of our case series also did not associate with epigenomic distinctness. Taken together, our data suggest that prominent perinuclear cytoplasmic CAM5.2 staining is not a crucial histopathological feature of DGST. Further investigations are needed to clarify if CAM5.2 immunoreactivity may associate with separate clinical features in DGST and whether CAM5.2-negative PitNETs with epigenomic profiles of SGST also exist.

### Are DGST-PIT1/SF1 truly plurihormonal tumors?

The question arises, how DGST-PIT1/SF1 should be meaningfully termed and classified. As previously mentioned, the WHO 2022 dictates to bundle all PitNETs with immunopositivity for more than one TF as “plurihormonal PitNET/adenoma”. Consequently, the WHO distinguishes DGST-PIT1/SF1 from pure PIT1-lineage somatotroph PitNETs and formally classifies these tumors together with various PitNETs exhibiting unusual combinations of TF and hormone expression patterns. Our data, however, suggests that DGST-PIT1/SF1 represents a distinct somatotroph PitNET subtype. Moreover, since DGST-PIT1/SF1 rarely expressed FSH or LH in our study, questioning the true plurihormonal identity of these tumors stands to reason. We propose the term “somatogonadotroph PitNET” for DGST-PIT1/SF1.

In conclusion, a substantial proportion of previously diagnosed somatotroph PitNETs co-express PIT1 and SF1 and exhibit clinical, histopathological, and molecular distinctness from other pure PIT1-lineage somatotroph PitNETs. We present a comprehensive meta-analysis of the three emerging molecular subtypes of somatotroph PitNETs, which call for a refinement of the current WHO 2022 classification.

### Supplementary Information

Below is the link to the electronic supplementary material.

Supplementary Figure 1: PIT1, SF1 and TPIT immunostaining of non-PIT1-lineage PitNETs and non-pituitary NETs. a-d) Gonadotroph PitNETs (n=30) stained with the antibodies PIT1, SF1 and TPIT used in this study stained exclusively and unequivocally for SF1. e-h) Corticotroph PitNETs (n=30) stained with the antibodies PIT1, SF1 and TPIT used in this study stained exclusively and unequivocally for TPIT. Scale bar is 50µm in b-d and f-h. i) Nearly all non-pituitary NETs were immunonegative for PIT1, SF1 and TPIT. In line with a recent study by Uccella et al. (2023), one NET of the lung (1/19) demonstrated moderate TPIT expression in roughly 5% of tumor cells (PNG 3921 KB)

Supplementary Figure 2: Intratumoral expression of prolactin, CAM5.2 immunonegativity or expression of gonadotropins does not indicate epigenomic distinctness among somatotroph PitNETs. a-f) Dimension reduction of global DNA methylation data via UMAP illustrates epigenomic similarities between PitNETs. Somatotroph PitNETs with evidence of intratumoral prolactin expression by immunostaining were not epigenomically distinct from somatotroph PitNETs without prolactin immunopositivity and did not affiliate with lactotroph PitNETs (d). Extent of perinuclear cytoplasmic CAM5.2 immunostaining was not associated with epigenomic distinctness among DGST. Of note, CAM5.2 immunonegative DGST did not separate from CAM5.2 immunopositive DGST (e). Among DGST-PIT1/SF1, expression of FSH or LH did not associate with epigenomic distinctness from FSH/LH immunonegative cases. Moreover, FSH/LH-expressing DGST did not affiliate with gonadotroph PitNETs (f). Shown plots in a – f are based on the beta values of the top 10,000 most variant CpGs (PNG 979 KB)

Supplementary Table 1: Overview of patient/sample characteristics of the in-house case series (XLSX 23 KB)

## Data Availability

The methylation data generated in this study are accessible via the GEO accession number GSE246645.
