# Supplementary figures and images for: Pituitary neuroendocrine tumors with PIT1/SF1 co-expression show distinct clinicopathological and molecular features
Source: Acta Neuropathol. 2024 Jan 16;147(1):16. doi: 10.1007/s00401-024-02686-1 (PMC10791732; doi:10.1007/s00401-024-02686-1)

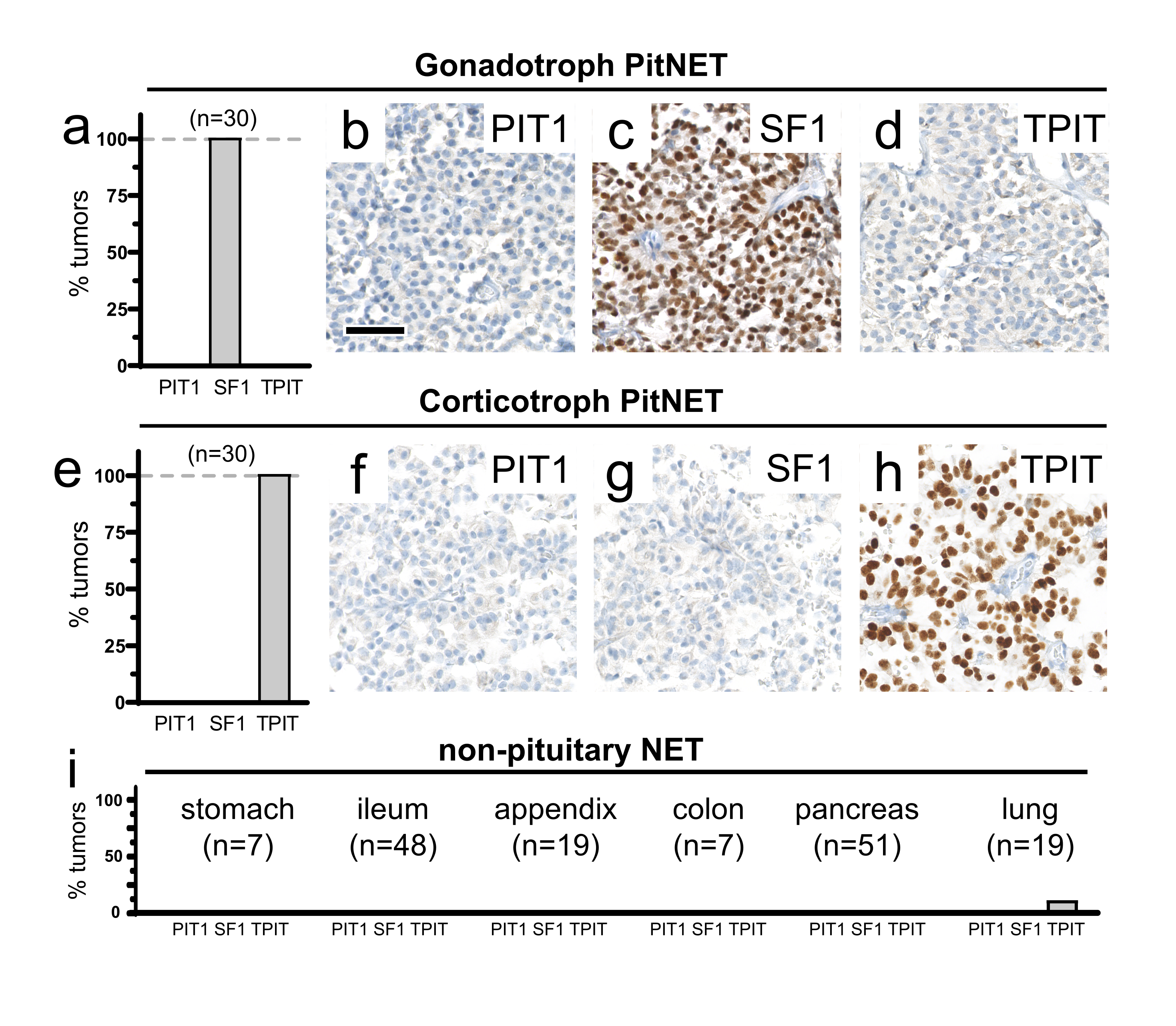

Supplement: Supplementary file 1 — Supplementary Figure 1: PIT1, SF1 and TPIT immunostaining of non-PIT1-lineage PitNETs and non-pituitary NETs. a-d) Gonadotroph PitNETs (n=30) stained with the antibodies PIT1, SF1 and TPIT used in this study stained exclusively and unequivocally for SF1. e-h) Corticotroph PitNETs (n=30) stained with the antibodies PIT1, SF1 and TPIT used in this study stained exclusively and unequivocally for TPIT. Scale bar is 50µm in b-d and f-h. i) Nearly all non-pituitary NETs were immunonegative for PIT1, SF1 and TPIT. In line with a recent study by Uccella et al. (2023), one NET of the lung (1/19) demonstrated moderate TPIT expression in roughly 5% of tumor cells (PNG 3921 KB) [file 401_2024_2686_MOESM1_ESM.png]

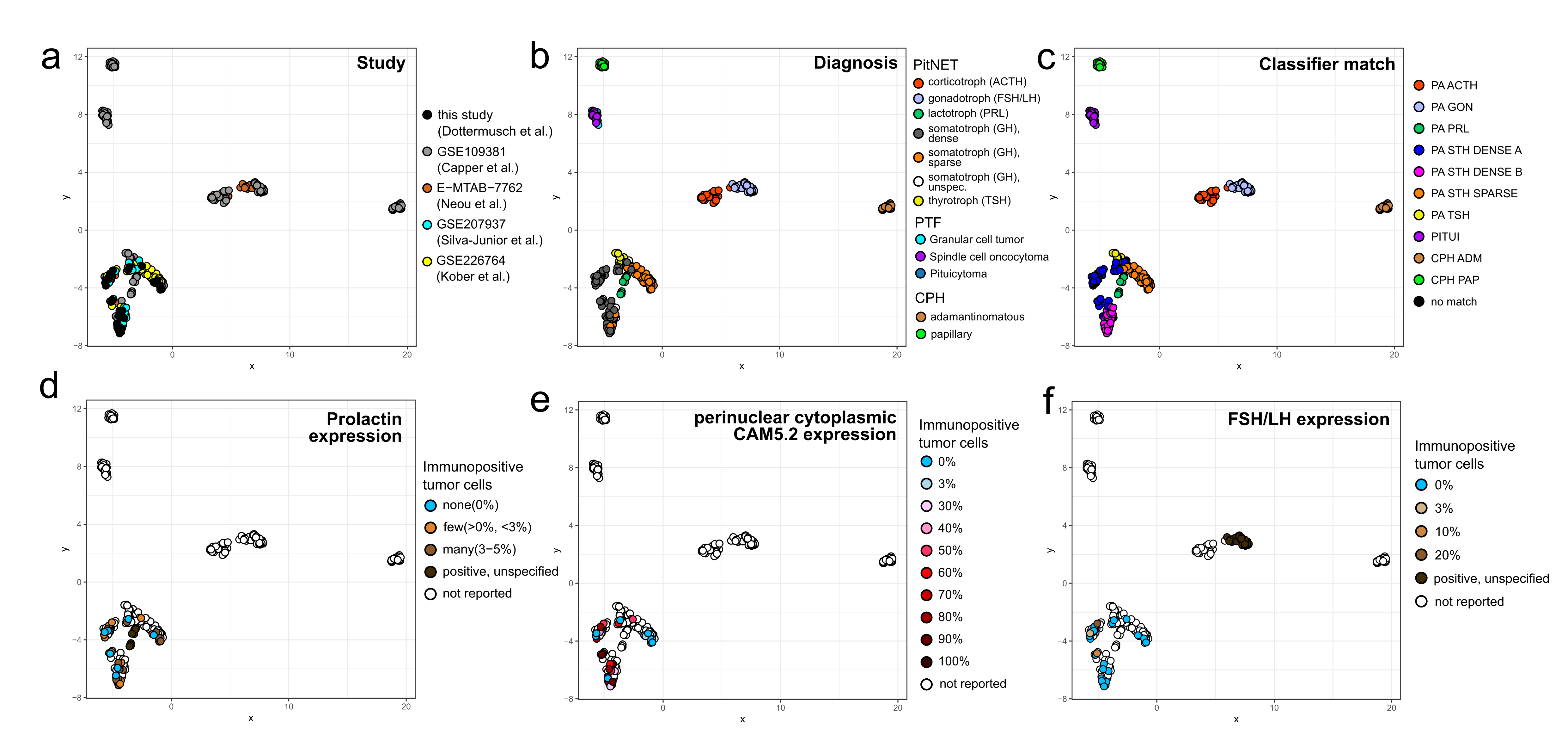

Supplement: Supplementary file 2 — Supplementary Figure 2: Intratumoral expression of prolactin, CAM5.2 immunonegativity or expression of gonadotropins does not indicate epigenomic distinctness among somatotroph PitNETs. a-f) Dimension reduction of global DNA methylation data via UMAP illustrates epigenomic similarities between PitNETs. Somatotroph PitNETs with evidence of intratumoral prolactin expression by immunostaining were not epigenomically distinct from somatotroph PitNETs without prolactin immunopositivity and did not affiliate with lactotroph PitNETs (d). Extent of perinuclear cytoplasmic CAM5.2 immunostaining was not associated with epigenomic distinctness among DGST. Of note, CAM5.2 immunonegative DGST did not separate from CAM5.2 immunopositive DGST (e). Among DGST-PIT1/SF1, expression of FSH or LH did not associate with epigenomic distinctness from FSH/LH immunonegative cases. Moreover, FSH/LH-expressing DGST did not affiliate with gonadotroph PitNETs (f). Shown plots in a – f are based on the beta values of the top 10,000 most variant CpGs (PNG 979 KB) [file 401_2024_2686_MOESM2_ESM.png]
